# Structural insights into the agonist activity of the nonpeptide modulator JR14a on C3aR

**DOI:** 10.1038/s41421-024-00765-x

**Published:** 2025-01-10

**Authors:** Ping Luo, Wenwen Xin, Shimeng Guo, Xin Li, Qing Zhang, Youwei Xu, Xinheng He, Yue Wang, Wenjia Fan, Qingning Yuan, Kai Wu, Wen Hu, Youwen Zhuang, H. Eric Xu, Xin Xie

**Affiliations:** 1 State Key Laboratory of Drug Research, Shanghai Institute of Materia Medica, Chinese Academy of Sciences, Shanghai, China; 2 University of Chinese Academy of Sciences, Beijing, China; 3 School of Pharmaceutical Science and Technology, Hangzhou Institute for Advanced Study, University of Chinese Academy of Sciences, Hangzhou, Zhejiang China; 4 Medicinal Bioinformatics Center, School of Medicine, Shanghai Jiao Tong University, Shanghai, China; 5 School of Life Science and Technology, ShanghaiTech University, Shanghai, China; 6 Shandong Laboratory of Yantai Drug Discovery, Bohai Rim Advanced Research, Institute for Drug Discovery, Yantai, Shandong China

**Keywords:** Cryoelectron microscopy, Autoimmunity

Dear Editor,

The complement system, a key component of innate immunity, remains inactive under normal conditions but activates in response to pathogens or antigen–antibody complexes, enhancing immune responses and maintaining tissue homeostasis^1^. Activation of the complement cascade produces anaphylatoxins like C3a and C5a, which regulate inflammatory and immune responses via their respective G protein-coupled receptors (GPCRs)^2,3^. Recent structural studies have elucidated the activation of these pathways^4,5^. Peptide ligands targeting C3aR are limited by their instability and administration route, hindering their therapeutic potential^6^, especially for the C3aR signaling-regulated neurodegenerative diseases and other disorders^7^. In contrast, small-molecule ligands offer enhanced stability and oral bioavailability, making them more useful for in vivo studies of C3aR and future clinical applications. However, only a few small-molecule ligands of C3aR, including SB290157, BR103 and JR14a, have been discovered. SB290157 and JR14a were reported as C3aR antagonists^8,9^, whereas BR103 is a full agonist^10^, despite their minimal structural differences (Supplementary Fig. S1a).

Nevertheless, several studies have suggested that both SB290157 and JR14a may act as C3aR agonists^11,12^. We also found that these compounds could activate C3aR across various pathways by functional experiments. JR14a showed dose-dependent inhibition of forskolin-induced cAMP in HEK293 cells expressing C3aR, demonstrating higher potency and efficacy in G_i_ activation compared to C3a (Fig. 1a). In human monocyte THP-1 cells, JR14a also showed dose-dependent inhibition of forskolin-induced cAMP production (Fig. 1b). Moreover, JR14a induced β-arrestin recruitment with similar potency but lower efficacy than C3a (Supplementary Fig. S1b). Additionally, JR14a induced C3aR internalization to a degree comparable to that of C3a after a 15-min treatment (Supplementary Fig. S1c). We also found that JR14a, SB290157 and BR103 could induce dose-dependent intracellular calcium elevation in HEK293 cells expressing C3aR and G_α16_ (Supplementary Fig. S1d), indicating that they all behave as C3aR agonists. C3aR activation can induce chemotaxis of neutrophils and monocytes^13^. Thus, we used the Transwell chemotaxis assay to evaluate the function of JR14a, with the chemokines fMLP and CCL2 as positive controls to induce neutrophil and monocyte migration, respectively. Both JR14a and C3a induced chemotaxis of mouse neutrophils and monocytes, further suggesting that JR14a is a C3aR agonist (Fig. 1c).

**Fig. 1:**
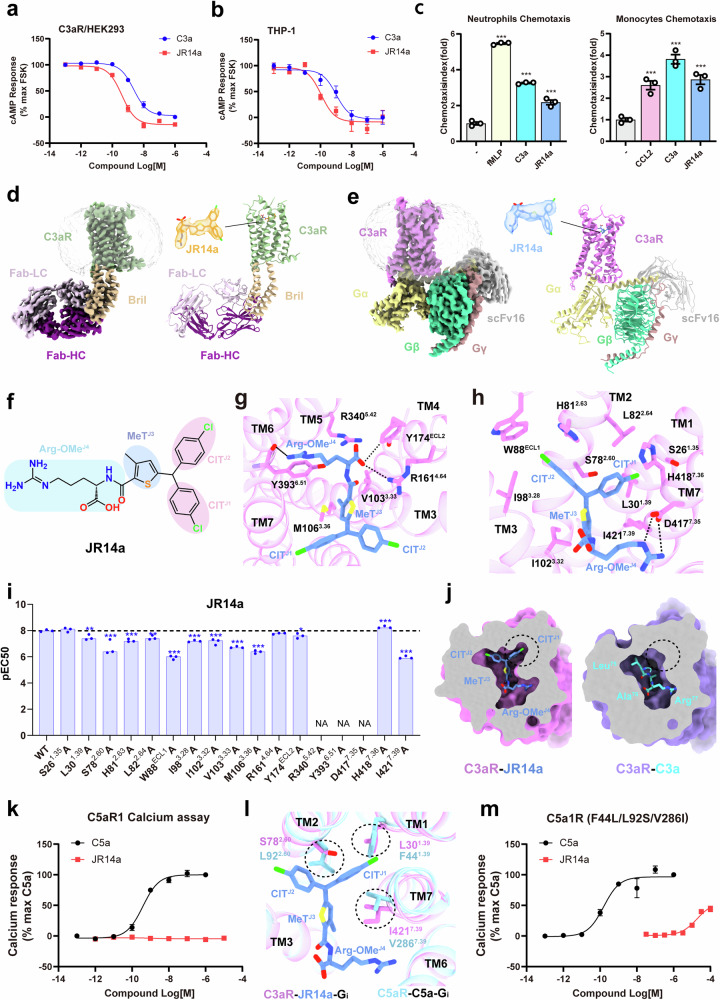
Structural and functional analysis of JR14a binding to C3aR. **a**, **b** Dose-dependent inhibition of forskolin-induced cAMP by C3a and JR14a in C3aR/HEK293 (**a**) and THP-1 cells (**b**). **c** Activity of chemokines and C3aR ligands in inducing chemotaxis of neutrophils and monocytes. ***P < 0.001. Significance was determined with one-way ANOVA followed by Fisher’s LSD multiple-comparison test, compared with the no treatment group. **d**, **e** Cryo-EM density maps and cartoon presentation of the C3aR–BRIL–JR14a–BAG2 complex (**d**) and C3aR–JR14a–G_i_ complex (**e**). In the C3aR–BRIL–JR14a–BAG2 complex, C3aR is colored in turquoise, BRIL in tan, JR14a in orange, BAG2 heavy chain (HC) in purple and light chain (LC) in thistle. In the C3aR–JR14a–G_i_ complex, C3aR is shown in violet, JR14a in blue, G protein complex in khaki (Gα), green (Gβ) and rosy brown (Gγ), and scFv16 in silver. **f** Illustration of the chemical structure of JR14a highlighting its group composition. **g**, **h** Interactions between JR14a and C3aR. **i** Potencies of JR14a towards C3aR mutants in the binding pocket measured with calcium release assay. **P < 0.01, ***P < 0.001. NA not activated. Significance was determined with one-way ANOVA followed by Fisher’s LSD multiple-comparison test, compared with the wild type. **j** Comparison of the binding pockets in C3aR–JR14a and C3aR–C3a (PDB 8HK2) complexes. **k** Measurement of C5aR1 activation induced by C5a and JR14a with calcium release assay. **l** Overlay of C3aR–JR14a–G_i_ and C5aR–C5a–G_i_ (PDB 8HK5) structures. **m** Activat_i_on of C5aR-F44L/L92S/V286I by JR14a measured with calcium release assay; C5a serves as a positive control. All functional assay data are presented as means ± SEM from at least three independent experiments.

Agonists can exhibit antagonist-like effects due to receptor desensitization, a common mechanism leading to the loss of GPCR functions after stimulation. For example, FTY720P is an agonist of S1PR, but its immunosuppressive function results from its functional antagonism of the receptor by inducing receptor desensitization and internalization^14^. In calcium assays, initial stimulation with C3a or JR14a induced robust calcium elevation in C3aR-expressing cells, while DMSO had no effect (Supplementary Fig. S1e, first arrow). Ten minutes later, re-stimulation with C3a only induced a calcium signal in cells pre-stimulated with DMSO, but not in those pre-stimulated with C3a or JR14a (Supplementary Fig. S1e, second arrow), indicating that JR14a and C3a induce similar features of receptor desensitization upon agonist stimulation.

To further explore the interaction between JR14a and C3aR, we conducted structural analysis of C3aR bound to JR14a, both with and without the heterotrimeric G_i_ protein. To solve the structure without G_i_, we used a common technique that involves substituting the ICL3 of C3aR with the BRIL protein in conjunction with an antibody Fab fragment, BAG2, to stabilize the C3aR–BRIL–JR14a complex. This complex structure was resolved at 3.0 Å (Fig. 1d; Supplementary Fig. S2 and Table S1). This method has been widely used to study GPCR structures in different states^15^. We also resolved the C3aR–JR14a–G_i_ complex structure at 2.9 Å (Fig. 1e; Supplementary Fig. S3). Both structures provided clear EM density maps, defining the binding poses of C3aR and JR14a.

These two C3aR–JR14a structures are similar to the previously reported C3aR–C3a–G_i_ structure, with root-mean-square deviation values less than 0.7 Å. The alignment of transmembrane domains and similar TM6 positions among the structures indicate that JR14a-bound C3aR adopted an active conformation, further supporting that JR14a is an agonist (Supplementary Fig. S4a).

JR14a primarily consists of two 4-chlorotoluene moieties (4-ClT^J1,2^), a 3-methylthiophene (3-MeT^J3^) and an arginine methyl ester (Arg-OMe^J4^) (Fig. 1f). It adopts a “λ“-shaped configuration within the orthosteric binding pocket of C3aR, with a buried surface area of 577 Å^2^, which is considerably smaller than that of polypeptide agonists (Supplementary Fig. S4b). Congruent three-dimensional alignments of JR14a with the C-terminal four residues of C3a and EP54 (a small peptide agonist of C3aR)^5^ indicate a similar binding mode across these peptide agonists. Specifically, JR14a aligns well with the last three amino acids of C3a and EP54, except for the ClT^J1^ (Supplementary Fig. S4c). However, from the fourth amino acid onwards, C3a and EP54 bind to the orthosteric pocket of C3aR in a hook-like shape, whereas JR14a remains flat within the bottom of the pocket (Supplementary Fig. S4b–d).

JR14a binds to an amphipathic pocket similar to the C-terminal residues of C3a (Supplementary Figs. S4e and S5a). The hydrophilic portion involves electrostatic interactions, hydrogen bonds and cation–π interactions, with Arg-OMe forming key contacts with D417^7.35^, R340^5.42^, and Y393^6.51^ (Fig. 1g, h). Arg-OMe also forms hydrogen bonds with Y174^ECL2^ and R161^4.64^ (Fig. 1g). Mutation of D417^7.35^, Y393^6.51^and R340^5.42^ to alanine directly abolished the activity of JR14a towards C3aR, whereas mutation of Y174^ECL2^ and R161^4.64^ to alanine had minimal impact (Fig. 1i; Supplementary Fig. S5b and Table S2). This indicates that Arg-OMe in JR14a is key for C3aR binding and activation, whereas ECL2 in C3aR is less critical for JR14a binding than for C3a^4,5^. The hydrophobic portion interacts with residues such as L82^2.64^, W88^ECL1^, I98^3.28^, I102^3.32^, V103^3.33^, M106^3.36^ and I421^7.39^(Fig. 1g, h); and mutating these residues, particularly W88^ECL1^and I421^7.39^, also led to a decrease in JR14a-mediated C3aR activation (Fig. 1i; Supplementary Fig. S5b). Notably, MeT^J3^ of JR14a resides in a hydrophobic environment without hydrogen bonding, which uniquely increases the interaction with I421^7.39^, thereby enhancing binding affinity (Fig. 1h).

Unlike C3a, JR14a occupies an additional minor pocket in the C3aR among TM1, TM2 and TM7 (Fig. 1j; Supplementary Fig. S4b). The ClT^J1^ of JR14a occupies this pocket and interacts with S26^1.35^, L30^1.39^, S78^2.60^, L82^2.64^ and I421^7.39^ to stabilize JR14a binding (Fig. 1h), eliminating the reliance on ECL2 and ECL3 for stabilizing the polypeptide ligand. Mutations of these residues, especially S78^2.60^ and I421^7.39^, significantly reduced the activity of JR14a in activating C3aR (Fig. 1i; Supplementary Fig. S5b). None of these mutations affected the expression of C3aR (Supplementary Fig. S5c).

The activation mechanisms of C3aR induced by JR14a and C3a are nearly identical; both involve the same structural rearrangements in the DRY, NPxxY, CWxP, and PIF motifs (Supplementary Fig. S6a, b), further confirming that JR14a functions as an activator of C3aR. Furthermore, molecular docking studies suggested that both SB290157 and BR103 bind to C3aR with a mode similar to that of JR14a (Supplementary Fig. S6c). Collectively, these studies confirmed that SB290157 and BR103, like JR14a, are agonists of C3aR.

Given the conserved binding mode of the C-termini of C5a and C3a, as well as C3a’s significant activity towards C5aR1^4^, it is plausible that JR14a would exhibit a similar effect towards C5aR1. Structural alignment demonstrates that JR14a adopts a nearly identical binding mode in different complement receptors, especially in the major pocket, compared with the last three C-terminal amino acids of C5a and C3a (Supplementary Figs. S4c, d, S5a and S7a). However, a subtle comparison between the configurations of JR14a and C5a reveals that in JR14a, the third-to-last residue, which is leucine in both C5a and C3a, is replaced by a chlorobenzene ring. Additionally, an extra ClT^J1^ extension fits into a unique binding cavity in C3aR that is absent in both C5aR1 and C5aR2. The binding sites within this minor pocket are not conserved across these receptors (Supplementary Fig. S7a). Consistent with this observation, JR14a did not show agonist activity towards C5aR1 or C5aR2 (Fig. 1k; Supplementary Fig. S7b, c), indicating that JR14a is a selective agonist for C3aR.

Further structural superimposition of the receptor components in the C5aR1–C5a and C3aR–JR14a complexes shows that all TMs align well. Two small residues L30^1.39^and S78^2.60^ are located within the extra pocket among TM1, TM2, and TM7 of JR14a-bound C3aR; their corresponding residues in C5aR1 are two larger residues F44^1.39^and L92^2.60^, which would cause significant steric hindrance with JR14a (Fig. 1l). Notably, the residue I421^7.39^ also plays a crucial role in JR14a binding to C3aR and its activation. When F44^1.39^, L92^2.60^, and V286^7.39^ in C5aR1 were mutated simultaneously to leucine, serine, and isoleucine, respectively, JR14a exhibited activity towards the mutated C5aR1 (Fig. 1m).

In summary, we resolved the first structure of C3aR binding to a small-molecule ligand JR14a, and clarified JR14a’s role as a C3aR agonist with both structural and functional evidence. JR14a occupies a unique cavity within C3aR, resembling the binding mode of peptide agonists, but with an additional interaction site among TM1, TM2, and TM7. This distinctive binding pocket, not conserved in related complement receptors, accounts for JR14a’s selective activation of C3aR. Our findings provide the first structural view of C3aR with a non-peptide agonist and offer a foundational basis for developing C3aR-specific small-molecule therapies for inflammatory diseases.

## Supplementary information

Supplementary Information

## Data Availability

The cryo-EM density maps of the C3aR–BRIL–JR14a–BAG2 and C3aR–JR14a–G_i_ complexes are available in the Electron Microscopy Data Bank under accession numbers EMD-60525 and EMD-60526, respectively. The atomic coordinates for these complexes are deposited in the Protein Data Bank under accession numbers 8ZWF and 8ZWG, respectively.

## References

[1] Ricklin, D., Hajishengallis, G., Yang, K. & Lambris, J. D. *Nat. Immunol.* **11**, 785–797 (2010). 20720586 10.1038/ni.1923 PMC2924908

[2] Merle, N. S., Church, S. E., Fremeaux-Bacchi, V. & Roumenina, L. T. *Front. Immunol.* **6**, 262 (2015). 26082779 10.3389/fimmu.2015.00262 PMC4451739

[3] Merle, N. S., Noe, R., Halbwachs-Mecarelli, L., Fremeaux-Bacchi, V. & Roumenina, L. T. *Front. Immunol.* **6**, 257 (2015). 26074922 10.3389/fimmu.2015.00257 PMC4443744

[4] Wang, Y. et al. *Nat. Chem. Biol.* **19**, 1351–1360 (2023). 37169960 10.1038/s41589-023-01339-w

[5] Yadav, M. K. et al. *Cell* **186**, 4956–4973.e21 (2023). 37852260 10.1016/j.cell.2023.09.020 PMC7615941

[6] Wilken, H. C., Gotze, O., Werfel, T. & Zwirner, J. *Immunol. Lett.* **67**, 141–145 (1999). 10232396 10.1016/s0165-2478(99)00002-4

[7] Bhatia, K., Ahmad, S., Kindelin, A. & Ducruet, A. F. *J. Clin. Invest.* **131** (2021). 10.1172/JCI144348 PMC7773380 33393493

[8] Ames, R. S. et al. *J. Immunol.* **166**, 6341–6348 (2001). 11342658 10.4049/jimmunol.166.10.6341

[9] Rowley, J. A. et al. *J. Med. Chem.* **63**, 529–541 (2020). 31910011 10.1021/acs.jmedchem.9b00927

[10] Reid, R. C. et al. *J. Med. Chem.* **57**, 8459–8470 (2014). 25259874 10.1021/jm500956p

[11] Mathieu, M. C. et al. *Immunol. Lett.* **100**, 139–145 (2005). 16154494 10.1016/j.imlet.2005.03.003

[12] Rodriguez, P. et al. *J. Biol. Chem.* **300**, 105549 (2024). 38072064 10.1016/j.jbc.2023.105549 PMC10796979

[13] Vandendriessche, S., Cambier, S., Proost, P. & Marques, P. E. *Front. Cell Dev. Biol.* **9**, 624025 (2021). 33644062 10.3389/fcell.2021.624025 PMC7905230

[14] Mullershausen, F. et al. *Nat. Chem. Biol.* **5**, 428–434 (2009). 19430484 10.1038/nchembio.173

[15] Guo, Q. et al. *Nat. Chem. Biol.* **20**, 74–82 (2024). 37580554 10.1038/s41589-023-01389-0

