# Supplementary material for: Structural insights into the agonist activity of the nonpeptide modulator JR14a on C3aR
Source: Cell Discov. 2025 Jan 10;11:7. doi: 10.1038/s41421-024-00765-x (PMC11718183; doi:10.1038/s41421-024-00765-x)
Supplement: Supplementary file 1 — Supplementary Information [file 41421_2024_765_MOESM1_ESM.pdf]

Supplementary Information

for

Structural Insights into the Agonist Activity of the Nonpeptide Modulator JR14a on C3aR

Ping Luo[1]†*, Wenwen Xin[1,2,3]†, Shimeng Guo[1], Xin Li[1,2], Qing Zhang[1], Youwei Xu[1], Xinheng He[1,2], Yue Wang[1], Wenjia Fan[1], Qingning Yuan[1], Kai Wu[1], Wen Hu[1], Youwen Zhuang[1,4], H. Eric Xu[1,2,5], Xin Xie[1,2,3,6]*

*Corresponding author: luoping@simm.ac.cn; xxie@simm.ac.cn

Materials and Methods

Preparation of C3aR-BRIL-JR14a-BAG2 and C3aR-JR14a-G_i complexes

The expression constructs for C3aR-BRIL were made by insert cytochrome b562 RIL (BRIL) in the intracellular loop 3 (ICL3). We also optimized the position of BRIL insertion based on the determined active C3aR structure to ensure the continuous connections between BRIL and the receptor. Finally, wild-type C3aR (residues 1-362 and 373-482) that is connected to BRIL using two short linkers derived from A2A adenosine receptor[1] was used for the structural determination. This construct was cloned with a N-terminal prolactin precursor sequence as a signal peptide, followed by FLAG-tag and a fragment of β2AR N-terminal tail region (BN) as fusion protein to increase the protein expression， and C termini was fused with His×8 tag into pFastbac vector.

The protein was expressed using baculovirus to infect Sf9 cells. After a 48-hour incubation at 27°C, the cells were harvested and resuspended in Buffer A (20 mM HEPES, pH 7.3, 100 mM NaCl, 10 μM JR14a, protease inhibitor cocktail). Membrane solubilization was then performed by adding 0.5% L-MNG and 0.1% CHS, then incubating at 4°C for 3 hours. The solubilized fractions were isolated by centrifugation at 100,000 g for 45 minutes. The resulting supernatant was incubated with Anti-FLAG M2 affinity agarose resin for 3 hours at 4°C. The resin was then washed with 20 column volumes (CV) of Buffer B (20 mM HEPES, pH 7.3, 100 mM NaCl, 0.01% LMNG, 0.002% CHS)). Elution was performed using 4 CV of Buffer B supplemented with 0.2 mg/mL FLAG peptide. To assemble the C3aR-BRIL-JR14a-BAG2 complex, the purified receptor was incubated with a 1.2-fold molar excess of BAG2 on ice for 1 hour. The synthetic anti-BRIL Fab BAG2 was expressed in *E. coli* and purified as described[2]. The mixture was then concentrated and further purified by size-exclusion chromatography using a Superdex 200 Increase 10/300 GL column in Buffer C (20 mM HEPES pH 7.3,100 mM NaCl, 10 μM JR14a, 0.00075% LMNG, 0.00025% GDN, 0.0002% CHS and 0.05% digitonin). Peak fractions were concentrated to approximately 12 mg/mL for electron microscopy studies.

The constructs of human C3aR were used to obtain JR14a-bound G_i complexes, following the same expression and purification procedures as in our previous study[3], with a modification to include 10 μM JR14a throughout all purification steps. The purified complex protein was concentrated and further purified by size-exclusion chromatography using a Superdex 200 Increase 10/300 GL column in Buffer C. Peak fractions were concentrated to approximately 15 mg/mL for electron microscopy studies. The purity of the complexes was evaluated through gel filtration and SDS-PAGE analysis.

Cryo-EM grid preparation and data acquisition

3.0 μL of purified 12 mg/mL C3aR-BRIL-JR14a-BAG2 complex was applied to a glow-discharged holey carbon EM grid (Quantifoil, Au 300 R1.2/1.3) in a Vitrobot chamber (FEI Vitrobot Mark IV). The C3aR-JR14a-G_i complex was also applied on carbon EM grid (Quantifoil, Au 300 R1.2/1.3). The chamber of the Vitrobot was set to 100% humidity at 4 $^{\circ}$C. The sample was blotted for 3s with a blot force of 2 and then plunged into liquid ethane. Cryo-EM data collection was taken by a Titan Krios (Thermo Fisher Scientific) using 300 kV accelerating voltage. The micrographs were recorded using a super-resolution counting mode at a pixel size of 0.824 Å using a K3 Summit direct electron detector (Gatan). Micrographs were obtained at a dose rate of about 15 $e^-/Å^2 \cdot s$ with a defocus ranging from −1.0 to −2.0 μm. A total movie with a total exposure time of 2.35 s for each micrograph was collected and was dose-fractionated to 36 frames, resulting in a total dose of ~ 50 $e^-/Å^2$.

Image processing and 3D reconstructions

All collected movie stacks were motion-corrected and dose-weighted in Relion4.0[4]. The contrast transfer function (CTF) parameters were estimated by CTFFIND4 program in CryoSPARC v.4.3.1[5,6].

For the C3aR-BRIL-JR14a-BAG2complex, auto-picking by Blob picker in CryoSPARC produced 3,637,923 particles that were subjected to 2D classification to generate input particles for Topaz train in CryoSPARC v.4.3.1. A total of 2,424,127particles were automatically picked by Topaz extract and were screened by several rounds of 2D classification in CryoSPARC v.4.3.1. Selected 1,597,205 particles were used to generate ab-initio map reference in CryoSPARC v.4.3.1, and subjected to a cascade of 3D classification and refinement in Relion4.0. To further improve the receptor structure in the complex. Local 3D classification with a receptor part 3D mask, the focused 3D classification resulted one good state, particles subjected to 3D refinement. After 3D refinement, particle polishing, CTF refinement and postprocess in Relion4.0, the resolution of structure for C3aR-JR14a-G_i complex reconstructed by 150,052 particles was 3.0 Å. The resolution was estimated by the gold-standard Fourier shell correlation (FSC)=0.143 criterion. Local resolution distribution was evaluated using Relion4.0.

For the C3aR-JR14a-G_i complex a similar procedure was applied and 122,627 good particles yielded reconstructions at 2.87 Å.

Cryo-EM model building and structure refinement

The cryo-EM structure of the C3aR-C3a-G_i complex (PDB: 8HK2) was used to build the model of C3aR-BRIL-JR14a-BAG2 and C3aR-JR14a-G_i complex. The model was fitted into the EM density map by UCSF Chimera, and manually adjusted in COOT based on the global EM map to build the main chain of C3aR and G_i, the GSK682753A-bound EBI2/GPR183 complex structure (PDB: 7TUY) was used as starting models for model building and refinement against the electron density map of BRIL and BAG2. Restraints for JR14a was generated based on SMILES string inputs using Phenix.elbow. The resulting models were refined against the EM density using real space refinement in PHENIX with secondary structure and geometry restrains. The final model after refinement was validated using the PHENIX package[7]. Extended Data Table 1 summarizes the model statistics. UCSF Chimera X was used to prepare the structural figures in the paper[8].

Molecular docking

The C3aR-JR14a complex structure was utilized for docking. Initially, C3aR was separated from the complex and prepared using the protein preparation wizard in Schrödinger's Maestro. Bond orders were assigned, and hydrogens were added to the protein. Disulfide bonds were created, and residue heteroatom states were defined using Epik at pH 7.0 ± 2.0. PROPKA was employed to assign residue protonation states. Grid files were generated based on the ligand binding pocket. Subsequently, BR103 and SB290157 were docked into these grids using the standard precision mode of the Glide program. The highest-scoring docking result was selected as the final outcome.

Plasmids construct for functional assay

Genes encoding wild-type C3aR, C5aR1 and C5aR2 were subcloned into the pcDNA3.0 vector with the addition of an N-terminal HA tag. All the mutations used for functional studies were generated by QuickChange PCR and were verified by DNA sequencing.

Cell culture and transfection

HEK293 cells were obtained from ATCC (Manassas, VA, USA) and cultured in DMEM supplemented with 10% (v/v) FBS, 100 mg/L penicillin, and 100 mg/L streptomycin in 5% CO_2 at 37 °C. For transient transfection, approximately 2.5×10^6 cells were mixed with 2 μg plasmids in 200 μL transfection buffer, and electroporation was carried out with a Scientz-2C electroporation apparatus (Scientz Biotech, Ningbo, China). The experiments were carried out 24 hours after transfection. HEK293 cell line stably expressing Gα16 was developed previously in our laboratory.

Calcium Mobilization Assay

Plasmids encoding C3aR, C5aR1 or C5aR2 were transfected into HEK293 stably expressing Gα16 and seeded at a density of 4×10^4 per well onto 96-well culture plates and incubated for 24 h at 37 °C in 5% CO_2. The culture medium was then changed to HBSS buffer (containing 0.5% BSA and 250

μM sulfinpyrazone) supplemented with 2 μM Fluo-4 AM and 0.05% Cremophor EL at 37 °C for 45 min. After removal of the excess dye, 50 μL HBSS buffer was added to the dye-loaded cells.

To test agonist activity, 25 μL HBSS buffer containing various concentration of testing compounds was added. The calcium change was measured using a FlexStation III microplate reader (Molecular Devices), and intracellular calcium change was recorded at an excitation wavelength of 485 nm and an emission wavelength of 525 nm. EC_{50} and E_{max} values for each curve were calculated by Prism 8.0 software (GraphPad Software).

To test receptor desensitization, dye loaded cells in 50 uL HBSS buffer were first stimulated with 25 μL HBSS buffer containing 1 μM C3a or JR14a, or vehicle (1% DMSO), and calcium changes were recorded. Then the cells were incubated at 37 °C for 10 min, the medium was changed to fresh HBSS buffer, and another 25 μL HBSS buffer containing 1 μM C3a was added. Then calcium change was measured.

cAMP assay

HEK293 cells expressing C3aR (C3aR/HEK293) were cultured in DMEM supplemented with 10% (v/v) fetal bovine serum and incubate in 5% CO_2 at 37 °C. THP-1 were cultured in RRMI 1640 supplemented with 10% (v/v) fetal bovine serum and incubate in 5% CO_2 at 37 °C.

For C3aR/HEK293, cells were harvested and re-suspended in DMEM containing 500 μM IBMX at a density of 2×10^5 cells/mL. Regarding THP-1, the cells were harvested and re-suspended in RRMI 1640 containing 500 μM IBMX at a density of 8×10^5 cells/mL. Subsequently cells were plated onto 384-well assay plates with 5 μL per well. Another 5 μL buffer containing 1 μM forskolin and compounds at various concentrations were added to the cells and the incubation lasted for 15 min at 37 °C. Intracellular cAMP levels were measured with a LANCE Ultra cAMP kit (PerkinElmer, TRF0264) and EnVision multiplate reader according to the manufacturer's instructions.

NanoBit for β-arrestin2 recruitment

The recruitment of β-arrestin2 to C3aR, C5aR1 or C5aR2 was measured using the Promega NanoBiT protein-protein interaction system. In brief, HEK293 cells seeded at 4×10^4 cells/well on 96-well plates were co-transfected with plasmids encoding LgBit-β-arrestin2 with C3aR-SmBit. For C5aR1 and C5aR2, the plasmids combination is SmBit-β-arrestin2 with C5aR1/C5aR2-LgBit. Twenty-four hours later, culture medium was replaced with 40 μL fresh culture medium without FBS. And 10 μL Nano-Glo Live Cell reagent was added according to the manufacturer's protocol (Promega, N2011) and the cells were incubated in a 37 °C, 5 % CO_2 incubator for 10 min. Then another 25 μL culture medium containing various concentrations of compounds were added to the cells. Bioluminescence was measured with an EnVision multiplate reader (PerkinElmer).

Receptor Internalization Assay

HEK293 cells expressing HA-tagged C3aR were seeded onto 96-well plate. Twenty-four hours later, culture medium were changed into fresh DMEM containing FITC-conjugated anti-HA antibody (sigma，H7411) and incubated for 30 min at 37 °C in 5% CO_2. After removal of excess antibody, cells were incubated in DMEM containing 1 μM C3a or JR14a, or vehicle (1% DMSO) for 15 min. Cells were then washed with PBS and fixed with 4% paraformaldehyde. After counterstaining of the nuclei with Hoechst 33342, fluorescent images were obtained with Operetta (PerkinElmer).

Chemotaxis Assay.

Mouse bone marrow cells were collected into RPMI 1640 containing 0.25% BSA at a density of 1×10^7 cells/mL. The cell suspensions (100 μL) were added in Transwell cups (filter pore size: 3 μm) inserted into the wells of the 24-well plate (Ultralow attachment; Costar, Corning, Acton, MA) containing buffer (300 μL/well) with chemoattractants (10 μM fMLP or 200 ng/mL CCL2), C3aR ligand (100 nM C3a or JR14a), or vehicle. The plates were transferred to an incubator (37 ℃,

atmospheric CO_2, humidified). After 1.5 h, the filter inserts were removed, the medium in the lower chamber was collected and cells were recovered by centrifugation. The cells were then incubated at 4 °C for 30 min with anti-mouse CD11b APC, anti-mouse Ly6G PE-Cy7 and anti-mouse Ly6C FITC antibodies. After through washing, the cells were analyzed by flow cytometry. Neutrophils and monocytes were counted through gates defined by $CD11b^{+}Ly6G^{+}$ cells (neutrophils) and $CD11b^{+}Ly6C^{+}$ cells (monocytes).

Cell surface expression measurements (ELISA)

HEK293 cells were transfected with various constructs and seeded onto 96-well plates. The next day, cells were washed with PBS, fixed with 4% PFA for 30 min, and then blocked with 2% BSA for 1 h. Cells were then incubated with anti-HA antibody (Sigma Aldrich, H6908) overnight at 4°C and then HRP-conjugated secondary antibody (Sigma Aldrich, 7074S) for 1 h at room temperature. Then cells were washed and incubated with 150 μL tetramethylbenzidine for 15 min before the reaction was stopped with 0.2 M H_2SO_4. Absorbance at 450 nm was quantified using a FlexStation III microplate reader (Molecular Devices). Determinations were made in triplicates.

Statistical analysis

All functional study data were analyzed with Prism 8 (GraphPad) and presented as means ± S.E.M. from at least three independent experiments. Concentration-response curves were evaluated with a three-parameter logistic equation. p*EC50* values were calculated using the sigmoid three-parameter equation. Significance was determined by one-way ANOVA followed by multiple comparisons test, and $*P < 0.05$ *vs*. wild-type (WT) was considered statistically significant.

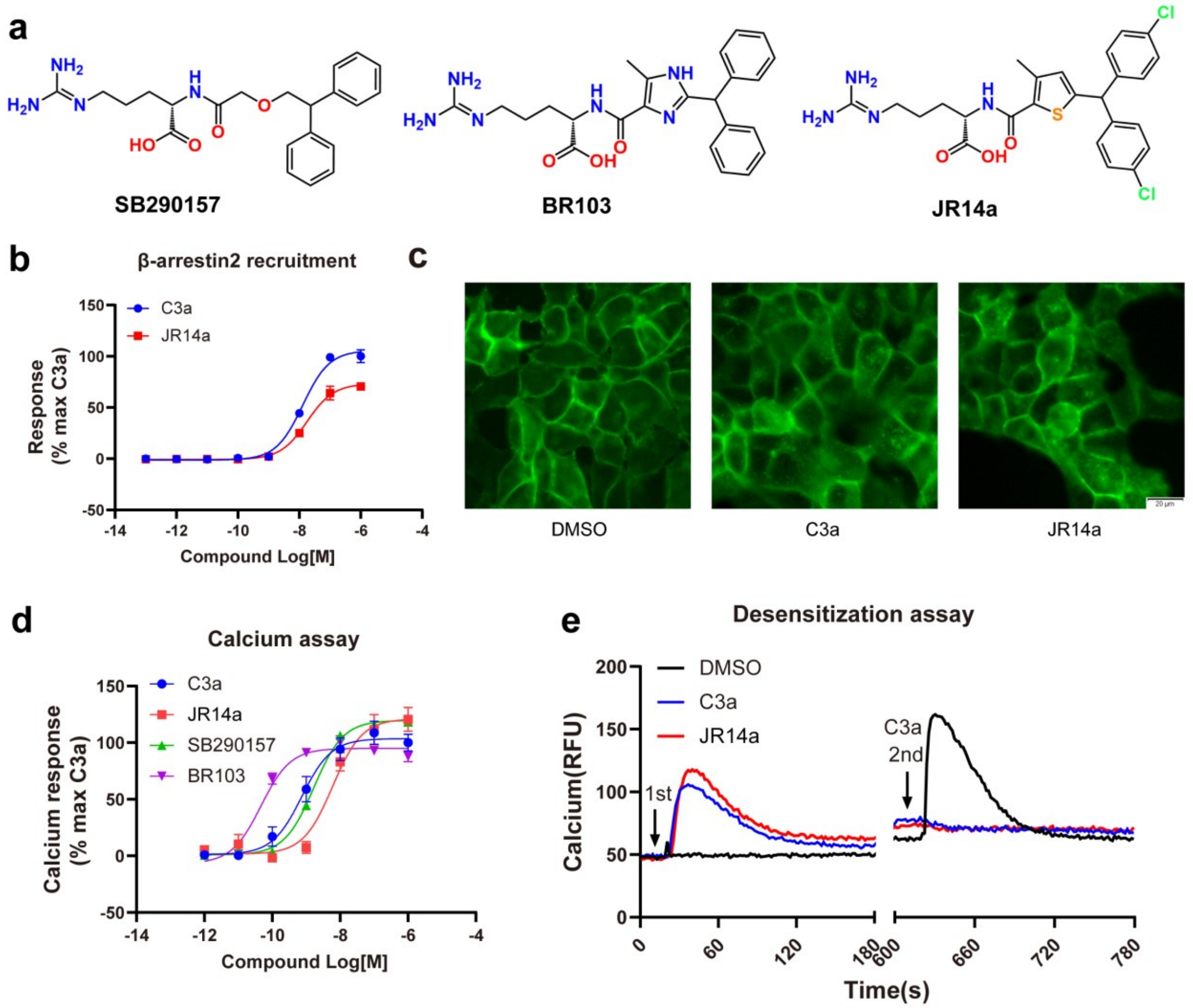

Supplementary Fig. S1 Chemical structures and functional analysis of C3aR modulators. a The Chemical structures of SB290157, BR103 and JR14a. **b** Concentration-response curves of C3a and JR14a in β-arrestin2 recruitment. **c** Representative fluorescence images of C3aR internalization in HEK293 cells expressing HA-tagged C3aR, stained with FITC-conjugated anti-HA antibody and stimulated with 1 μM C3a, JR14a, or vehicle (1% DMSO) for 15 minutes, and then fixed. **d** Concentration-response curves of C3a, JR14a, SB290157 and BR103 in inducing intracellular calcium release in HEK293 cells expressing C3aR and $G_{\alpha16}$. Data are mean ± SEM from three independent experiments. **e** Receptor desensitization measured with calcium assay. HEK293 cells expressing C3aR and $G_{\alpha16}$ were first stimulated with vehicle (1% DMSO), C3a or JR14a (1 μM), and calcium changes were recorded (1st arrow). Then the cells were incubated at 37 °C for 10 min, the medium was changed to fresh HBSS buffer, and then stimulated with 1 μM

C3a (2nd arrow). Data are means ± SEM from three independent experiments.

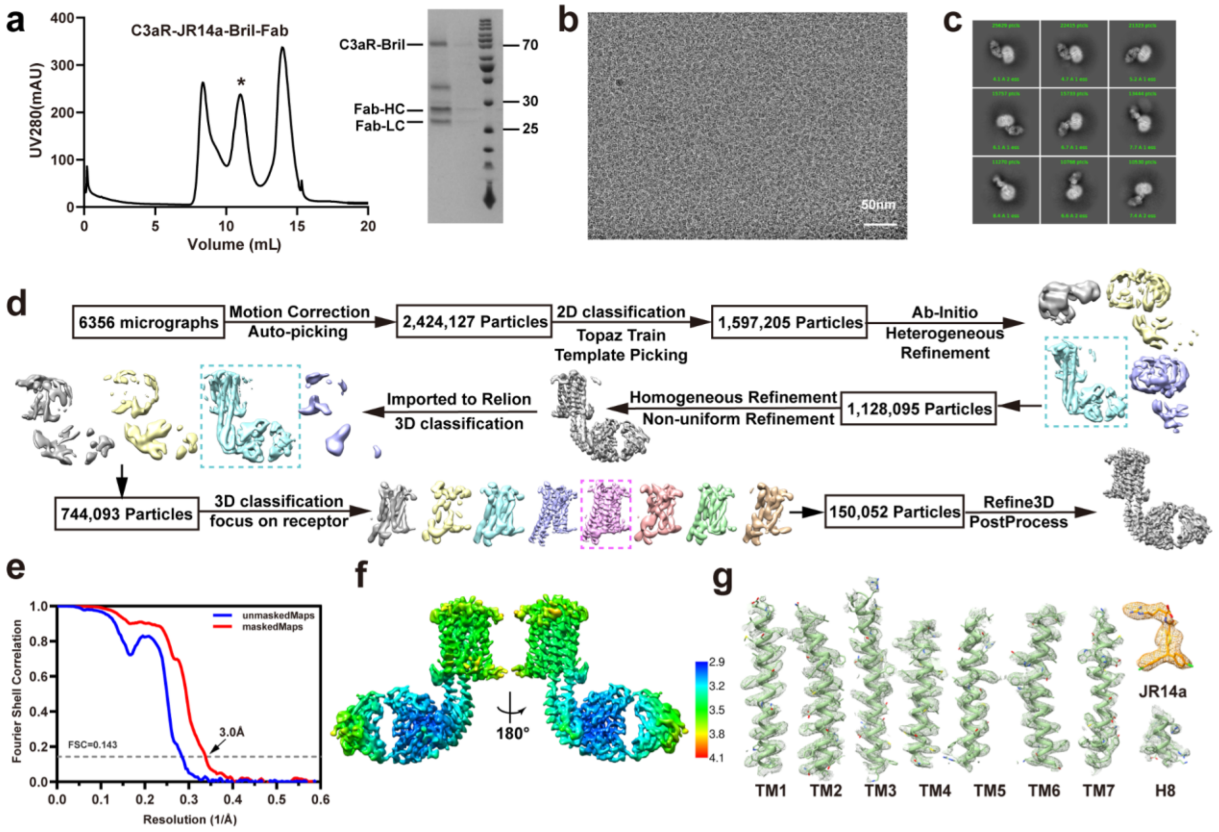

Supplementary Fig. S2 Cryo-EM structure determination of C3aR-BRIL-JR14a-BAG2 complex. a Size-exclusion chromatography elution profile (left) and SDS-PAGE analysis (right) of the C3aR-BRIL-JR14a-BAG2 complex. **b** Representative cryo-EM micrograph. **c** 2D class averages. **d** Cryo-EM data process flowchart of C3aR-BRIL-JR14a-BAG2 complex by CryoSPARC v.4.3.1 and Relion4.0. **e** The Fourier shell correlation (FSC) curve at 0.143 of the processed density map indicate an estimated resolution of 3.0 Å. **f** Final cryo-EM map surface colored to show the local resolution estimated by Relion4.0. **g** Density maps of helices, and JR14a**.**

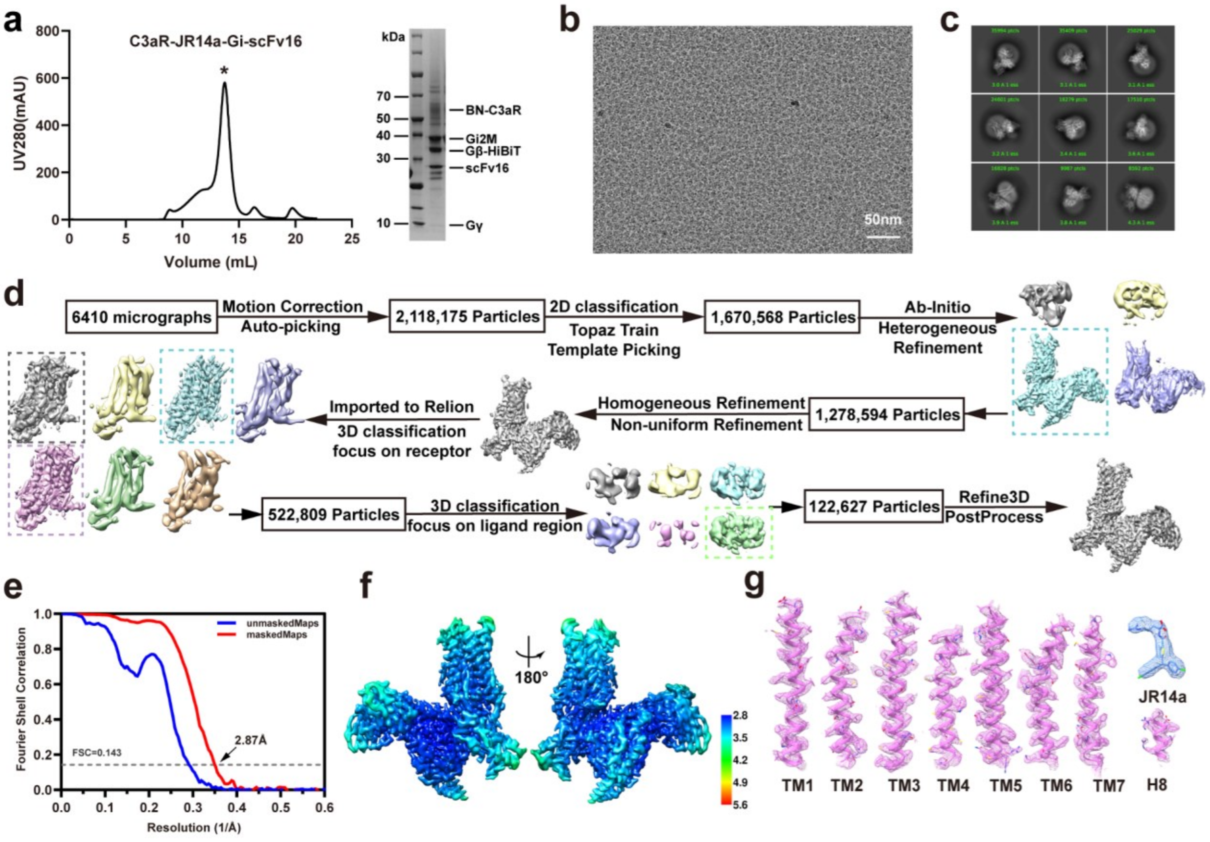

Supplementary Fig. S3 Cryo-EM structure determination of C3aR-JR14a-G_i complex. a Size-exclusion chromatography elution profile (left) and SDS-PAGE analysis (right) of the C3aR-JR14a-G_i complex. **b** Representative cryo-EM micrograph. **c** 2D class averages. **d** Cryo-EM data process flowchart of C3aR-JR14a-G_i complex by CryoSPARC v.4.3.1 and Relion4.0. **e** The Fourier shell correlation (FSC) curve at 0.143 of the processed density map indicate an estimated resolution of 2.87 Å. **f** Final cryo-EM map surface colored to show the local resolution estimated by Relion4.0. **g** Density maps of helices, and JR14a**.**

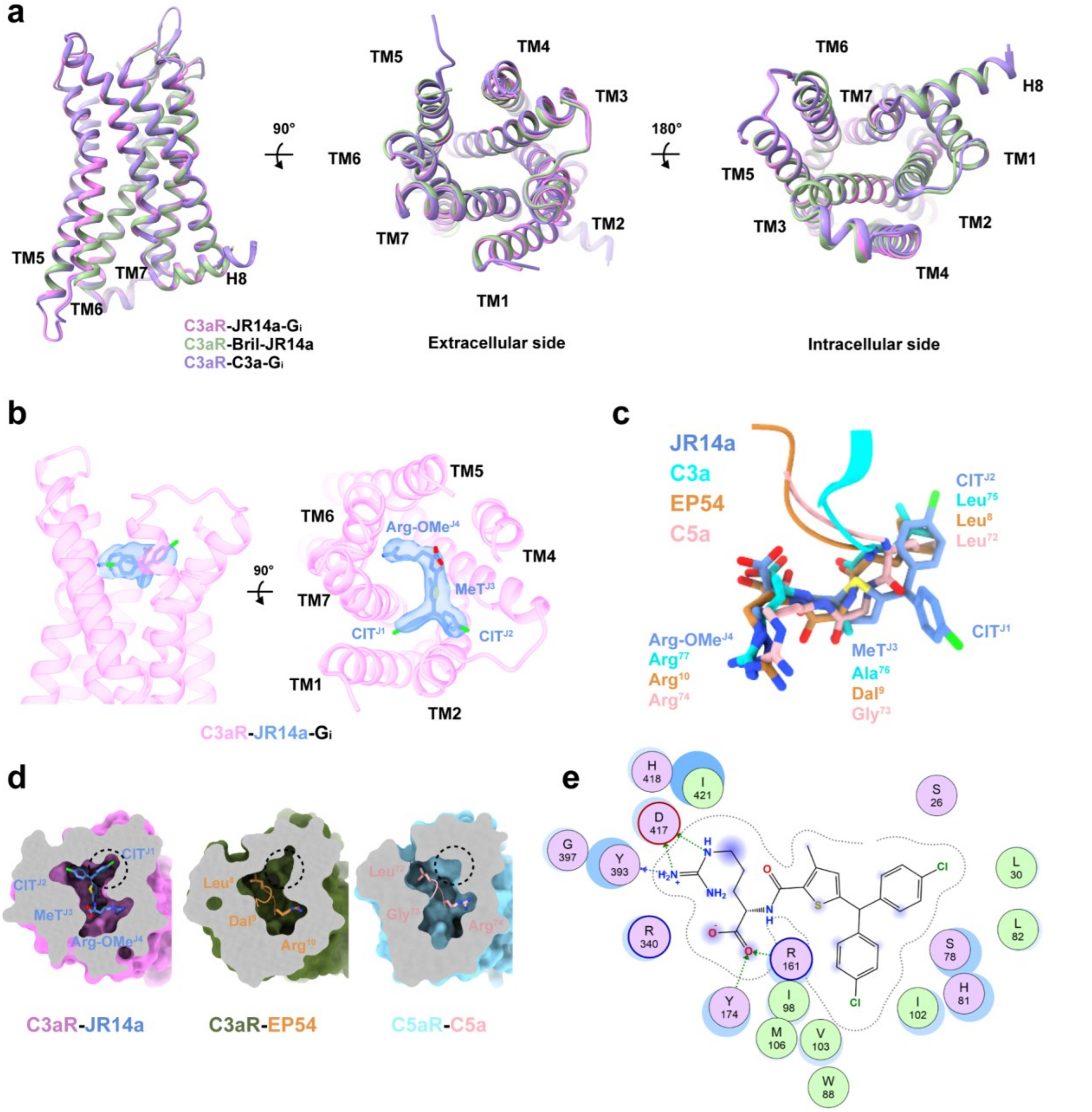

Supplementary Fig. S4 Structural comparison and interaction analysis of C3aR bound to JR14a. **a** Overlay of C3aR-JR14a-G_i (violet), C3aR-BRIL-JR14a (turquoise) and C3aR-C3a-G_i (PDB:8HK2, light purple) structures. **b** Localization of JR14a within C3aR. **c** Comparison of ligands in orthosteric pocket on complement receptors**,** JR14a (blue); C3a (PDB:8HK2, sky blue); EP54 (PDB:8I95, brown); C5a (PDB:8HK5, pink). **d** Compare of the binding pockets. The receptor is shown as surface. **e** 2D C3aR-JR14a interaction diagram generated using the MOE software. non-polar and polar residues are presented in green and pink colors; green-dashed arrow

represents hydrogen bonding interaction; arene-hydrogen bonding interactions are shown with green colors; solvent accessible surface area for ligand and receptor is shown with blue smudge and turquoise halo; the shape of the binding site indicated by the proximity contour (dotted lines) surrounding the ligand.

a

	1.35	1.39	2.60	2.63	2.64	ECL1	ECL1	3.28	3.29	3.32	3.33	3.36	4.60	4.64	ECL2	5.42	6.51	6.55	7.35	7.36	7.39
JR14a	S26	L30	S78	H81	L82	-	W88	I98	-	I102	V103	M106	-	R161	Y174	R340	Y393	G397	D417	H418	I421
C3a	-	-	S78	H81	-	-	-	-	P99	I102	V103	-	V157	R161	Y174	R340	Y393	G397	D417	H418	I421
EP54	-	-	S78	H81	-	-	W88	-	P99	I102	V103	M106	-	R161	Y174	R340	Y393	G397	D417	-	I421
C5a	-	-	L92	-	I96	H100	-	-	P113	I116	L107	M120	-	R175	V190	R206	Y258	G262	D282	S283	-

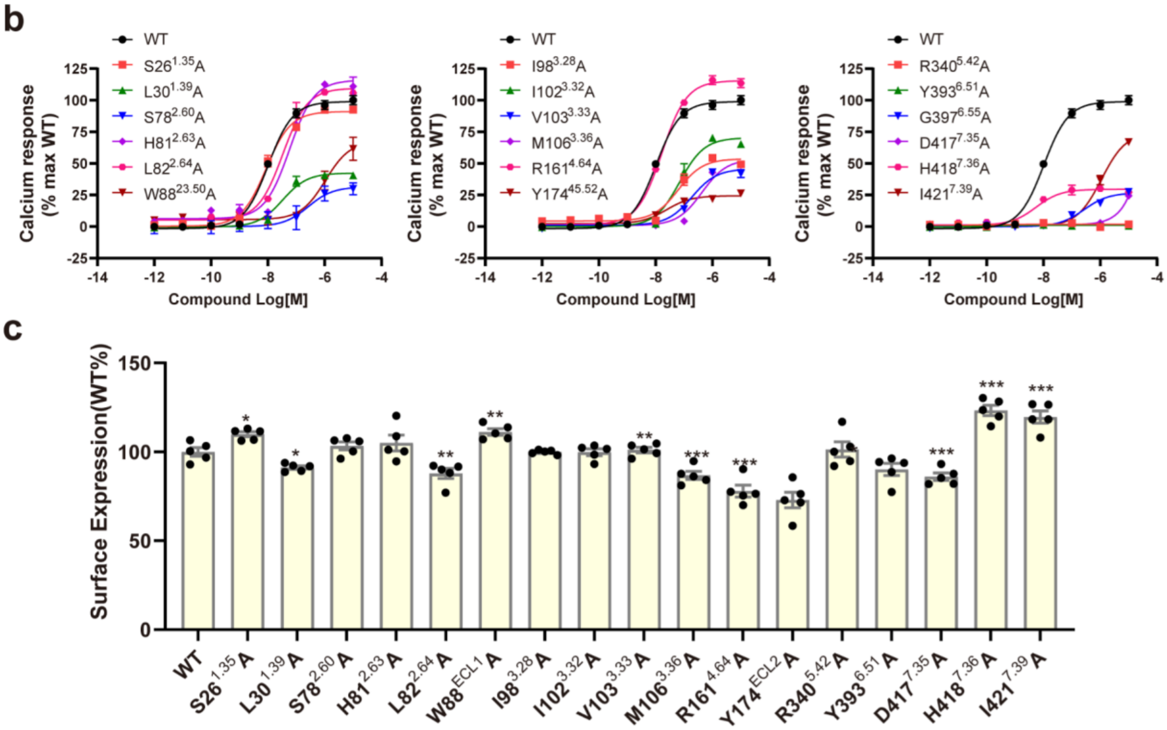

Supplementary Fig. S5 Analysis of the binding pocket of JR14a. a Alignment of the binding site sequences in complement receptors for each ligand. **b** Concentration-response curves of JR14a in activating C3aR carrying mutations in the binding pocket measured with calcium release assay. Data shown are means ± SEM from at least three independent experiments. **c** Surface expression of the C3aR mutants.

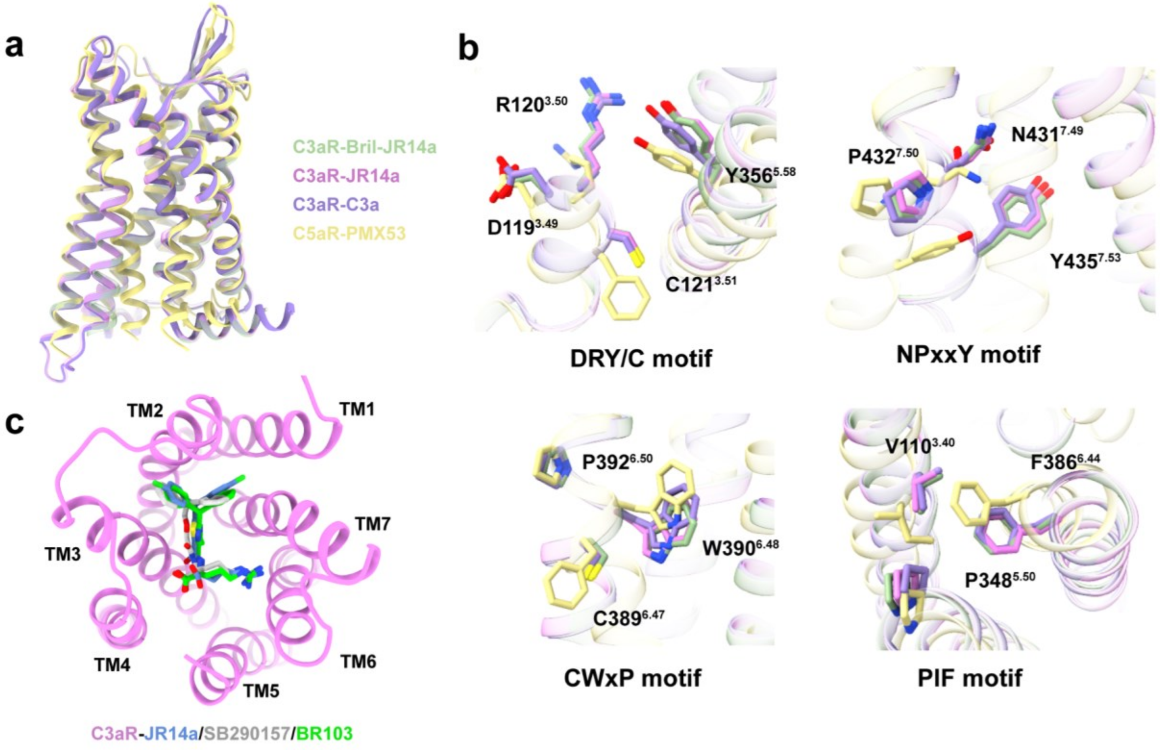

Supplementary Fig. S6 Analysis of the JR14a activation mechanism. a Overlay of C3aR-BRIL-JR14a (turquoise), C3aR-JR14a (violet), C3aR-C3a (PDB:8HK2, light purple) and C5aR-PMX53 (PDB:6C1R, yellow) structures. **b** Comparison of crucial residues and motifs involved in C3aR activation, encompassing the DRY/C, NPxxY, CWxP and PIF motif. **c** Superposition of JR14a, SB290157, and BR103 in C3aR ligand pocket, coordinates of SB290157 (gray) and BR103 (green) were determined by molecular docking.

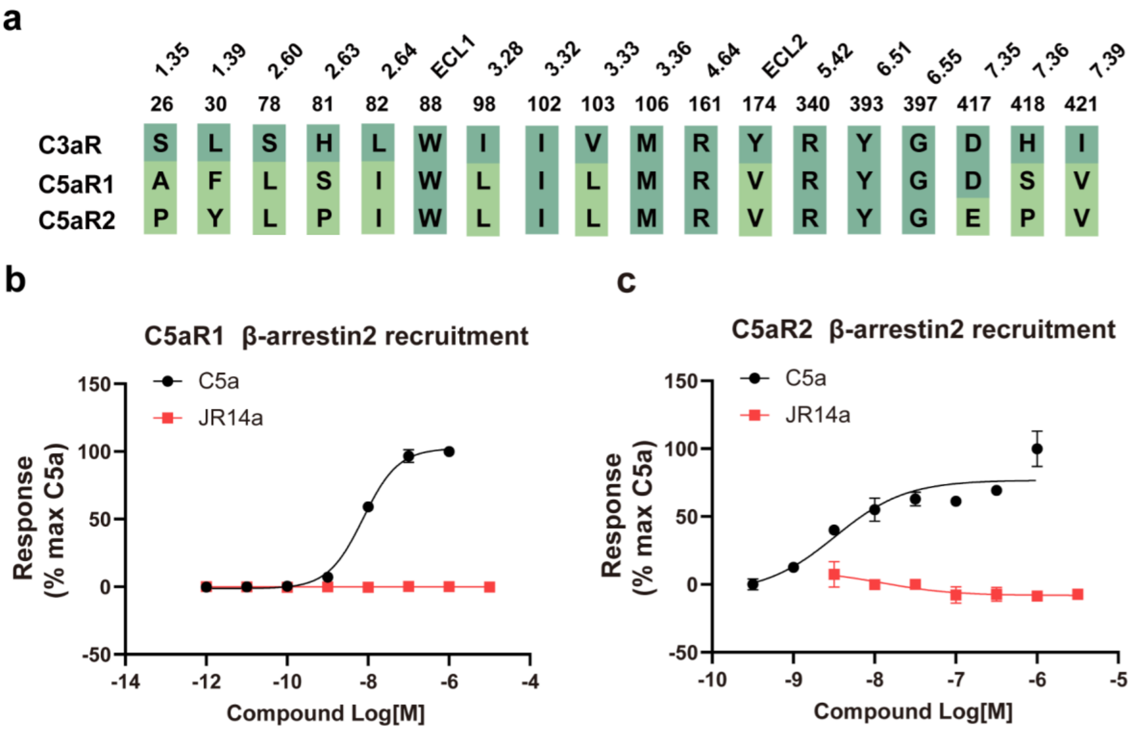

Supplementary Fig. S7 JR14a does not exhibit activity towards C5aR1 and C5aR2. a Comparison of the residues in the JR14a binding site within C3aR with those in C5aR1 and C5aR2. **b, c** Measurement of the activation of C5aR1(b) and C5aR2 (c)by C5a and JR14a with β-arrestin recruitment assays. Data are mean ± SEM from three independent experiments.

Supplementary Table S1 Cryo-EM data collection, model refinement and validation statistics.

	C3aR_JR14a_G_i protein complex	C3aR_JR14a_BRIL complex
**Data collection and processing**		
Detector	K3	K3
Magnification	105,000	105,000
Voltage (kV)	300	300
Electron exposure ($e^-/Å^2$)	50	50
Defocus range (μm)	-1.0~-3.0	-1.0~-3.0
Pixel size (Å)	0.824	0.824
Symmetry imposed	C1	C1
Initial particle projections (no.)	2,118,175	2,424,127
Final particle projections (no.)	122,627	150,052
Map resolution (Å)	2.87	3.0
Map resolution range (Å)	2.82-4.89	2.98-4.41
FSC threshold	0.143	0.143
**Model Refinement**		
Refinement package	PHENIX-1.17.1-3660	PHENIX-1.17.1-3660
Real or reciprocal space	Real space	Real space
Model-Map CC (mask)	0.76	0.45
Model resolution (Å)	3.32	4.10
FSC threshold	0.5	0.5
B factors ($Å^2$, min/max/mean value)		
Protein residues	30.00/119.04/74.57	0.12/121.28/58.44
Ligands	15.26/15.26/15.26	15.26/15.26/15.26
**Model composition**		
Non-hydrogen atoms	8,709	6,206
Protein residues	1,121	801
R.m.s. deviations		
Bond lengths (Å)	0.004	0.003
Bond angles (°)	0.888	0.538
**Validation**		
MolProbity score	1.32	1.41
Clashscore	5.90	5.08
Rotamer outliers (%)	0.21	0.29
Ramachandran plot		
Favored (%)	99.18	97.21
Allowed (%)	0.82	2.79
Disallowed (%)	0.00	0.00
**Data availability**		
EMDB entry	EMD-60526	EMD-60525
PDB entry	8ZWG	8ZWF

Supplementary Table S2 Cell surface expression of C3aR and pEC_{50} values of JR14a on WT or mutant C3aR.

	Calcium assay		Cell-surface expression [a]
	pEC_{50} [a]	Emax [a]	
WT	7.98±0.05	100±3.6	100±2.4
S26$^{1.35}$A	8.06±0.09	92.6±2.5	110±1.4*
L30$^{1.39}$A	7.47±0.12**	40.5±0.29***	91.2±0.96*
S78$^{2.60}$A	6.67±0.32***	29.9±4.8***	103±2.2
H81$^{2.63}$A	7.23±0.07***	111±7.2	105±4.4
L82$^{2.64}$A	7.45±0.09**	106±0.92	87.9±2.8**
W88$^{23.50}$A	5.95±0.08***	61.5±9.1***	111±1.8**
I98$^{3.28}$A	7.19±0.04***	49.3±2.7***	99.9±0.53
I102$^{3.32}$A	7.15±0.13***	65.2±1.8***	99.8±1.9
V103$^{3.33}$A	6.73±0.04***	41.9±3.1***	101±1.5
M106$^{3.36}$A	6.37±0.07***	48.7±2.1***	86.8±2.3**
R161$^{4.64}$A	7.77±0.03	114±3.5	78.0±3.3***
Y174$^{45.52}$A	7.59±0.11*	26.3±0.58***	72.9±4.4***
R340$^{5.42}$A	NA [b]	1.79±1.0***	101±4.4
Y393$^{6.51}$A	NA	0.60±2***	90.1±3.4*
D417$^{7.35}$A	<5	24.4±0.80***	86.1±2.1***
H418$^{7.36}$A	8.26±0.05***	27.4±2.6***	123±2.9***
I421$^{7.39}$A	5.95±0.06***	66.9±2.0***	120±3.4***

[a] Data shown are means ± S.E.M. from at least three independent experiments.

[b] NA indicates that the activation level is too low to determine pEC_{50} values.

* $P<0.01$; $^{**}P<0.001$ and $^{***}P<0.0001$ by one-way ANOVA followed by multiple comparisons test, compared with WT.

References

1 Chen, H., Huang, W. & Li, X. Structures of oxysterol sensor EBI2/GPR183, a key regulator of the immune response. *Structure* **30**, 1016-1024 e1015 (2022). https://doi.org:10.1016/j.str.2022.04.006

2 Mukherjee, S. *et al.* Synthetic antibodies against BRIL as universal fiducial marks for single-particle cryoEM structure determination of membrane proteins. *Nat Commun* **11**, 1598 (2020). https://doi.org:10.1038/s41467-020-15363-0

3 Wang, Y. *et al.* Revealing the signaling of complement receptors C3aR and C5aR1 by anaphylatoxins. *Nat Chem Biol* **19**, 1351-1360 (2023). https://doi.org:10.1038/s41589-023-01339-w

4 Xu, K. *et al.* EZH2 oncogenic activity in castration-resistant prostate cancer cells is Polycomb-independent. *Science* **338**, 1465-1469 (2012). https://doi.org:10.1126/science.1227604
5 Martin, C., Cao, R. & Zhang, Y. Substrate preferences of the EZH2 histone methyltransferase complex. *The Journal of biological chemistry* **281**, 8365-8370 (2006). https://doi.org:10.1074/jbc.M513425200
6 Chang, C. J. & Hung, M. C. The role of EZH2 in tumour progression. *British journal of cancer* **106**, 243-247 (2012). https://doi.org:10.1038/bjc.2011.551
7 Adams, P. D. *et al.* Recent developments in the PHENIX software for automated crystallographic structure determination. *J Synchrotron Radiat* **11**, 53-55 (2004). https://doi.org:10.1107/s0909049503024130
8 Pettersen, E. F. *et al.* UCSF ChimeraX: Structure visualization for researchers, educators, and developers. *Protein Sci* **30**, 70-82 (2021). https://doi.org:10.1002/pro.3943
